# Supplementary material for: Occupational Safety, Health, and Well-Being Concerns and Solutions for Management Reported by Sign Language Interpreters: A Qualitative Study
Source: Int J Environ Res Public Health. 2024 Oct 23;21(11):1400. doi: 10.3390/ijerph21111400 (PMC11594195; doi:10.3390/ijerph21111400)
Supplement: Supplementary file 1 [file ijerph-21-01400-s001.zip › ijerph-3194280 supplementary/ijerph-3194280 data set.pdf]

Domains n=27	Listening session 1	Listening session 2	Listening session 3	Listening session 4	Listening session 5	Listening session 6	Listening session 7	Listening session 8
	**December 13, 2023**	**January 3, 2024**	**January 19, 2024**	**February 12, 2024**	**February 23, 2024**	**March 1, 2024**	**March 20, 2024**	**March 22, 2024**
	**n=4**	**n=4**	**n=4**	**n=4**	**n=4**	**n=3**	**n=2**	**n=2**
**Mental health concerns/strategies**	"I kind of go in these waves where I'm really good, I'm feeling like a team, if we can kindof see each other or we get together and then, sometimes, I feel, like super just like isolated. Like, I have no idea what anybody else is doing. I don't know and you know, it doesn't feel good. Like, we have these students and we just don't see them very often and we don't get to mentor them as much as I would like to and so that's a concern, well, what, where are these other people that we are working with **(Interpreter #4)?"**<br><br>"I've been participating in supervision sessions, which has actually been super beneficial to like, my mental health and well-being. Being able to discuss and talk about the work is a really productive way **(Interpreter #4)…**"<br><br>"For freelance interpreters, in Indiana specifically, there is not really an outlet for interpreters to get together and discuss in a confidential way, things that are going on or situations that might arise versus	"I have chosen to not do VRI or VRS work because to me it is super stressful. I've heard stories. I have friends that do that work. The demands that are put on you. I want to be able to control what I'm walking in to and whenever I'm going into a VRI or VRS thing, during COVID, you know, we were virtual for school. That was just so stressful for me and again, we're here to provide a service and we do it because we love what we do. I never kindof came into this profession, thinking oh, I'm going to make so much money." "I'm not gonna put myself out there to just go and make more money because you know? I don't know, that's… my mental health is more important than that **(Interpreter #5)**."<br><br>Regarding this new set of demands since COVID, "as human beings, I'm noticing interpreters, my own colleagues, are far more afraid than they were in the past. I've noticed there's a higher	The job is lonely and I have to find ways to support myself through difficult assignments or interactions. I think cause I've lasted so long at doing this, I must be able to do that well enough that I haven't had a crisis of not wanting to continue as an interpreter. But it's not a given. I definitely have to have the space to remind myself what was hard, remind myself that I can debrief and I should debrief and I can talk to trusted colleagues **(Interpreter #11).**<br><br>Some of those things you have to be able to digest them with someone else and be like, am I looking at this weird? Or, just like, can I just tell you what happened today? Like, I've gotta get this out and then, kindof like putting it out there is like okay, I feel better, I don't have to wear this and that emotional stress affects your physical health **(Interpreter #12)**.<br><br>Because of the way that our industry is and because of the system that we operate within, we're forced to make choices that it's like a bad choice or worse choice. Right?	Part of what contributes to mental health and freelance interpreters and longevity in the field, is understanding it's not a 9a-5p, it's not a job where you have a boss who tells you what to do and you have choice to take a break when you need to without fear of losing your job **(Interpreter #13)**.<br><br>The other part that came up for me right away was that the Total Worker Health®, this whole kindof new concept for me feels not so much connected to the physical strain of the work but the emotional and psychological demands and I feel like that's the thing that I have seen most **(Interpreter #15)**.<br><br>When people get over that [the physical part or mechanics of interpreting] or perhaps when they work in situations where they have teams and the work environment is less demanding and more forgiving in terms of time and	I talked about doing some work outside of interpreting and one of the things I did and it's probably been 15 years ago now was, I was fortunate to participate in a workshop that forced me to really, like you mentioned [Interpreter #20], who am I outside of interpreting? I'm not an interpreter. Interpreting is what I do to earn a living, but I'm so much more than an interpreter and so I had to really take a look at who am I? What am I? If I am not interpreting, what else would I be? What else could I do? And so, I have other outlets, as well, like you [Interpreter #20] mentioned. I'm a pet Mom. I love to play in the kitchen. I love to write, so I have all of these other options that if I had to give up interpreting, I wouldn't feel like I was, like I'm dead now. I have nothing left. I have all of these other interests that I can pursue. Who are you outside of being an interpreter, I think, is really, really important **(Interpreter #19)**.<br><br>Completely agree with that [giving people the space to explore], like what is and that's what I was saying, what's missing? When you're looking at interpreting is too overwhelming, the pieces that are missing are the pieces of yourself that you're not giving	During COVID, when you couldn't get in to see a medical provider, you couldn't get into the see a vet, like a specialty vet because they were limiting their work and still a lot of the vets and medical professionals left, but now, it's like 2-3 months to get into an appointment and you're like, yea, but I'm suffering now, they're like your next appointment is 3 months. I wish we could a little bit take something from that because they're definitely using self-care. They are saying, I have 8 hours to my day, I have this many medical slots for appointments and the next available slot is in 3 months, do you what it? And we're like, okay, so maybe I can fit you in, you know? We're not there, maybe we might learn something **(Interpreter #23)**.<br><br>When I get a request for an interpreting assignment, I will	Interpreters, it didn't matter if they were hearing or deaf, with limited extra-linguistic knowledge (ELK), they don't know the topic, seem more likely to stay with the form. And I see more stress and processing… interesting, I saw for myself, they seem more stressed while signing because they don't understand that information. They don't feel connected with their whole body. They say that know the topic, that it's easy, that they can use their prediction skills, that they can process, but it's more of the same form. They fingerspell more, so that can have an impact. Compared to the interpreter who knows that topic, more free form, and can figure out how to sign. Based on my observation, I wouldn't be surprised if they hurt less; specifically, interpreters who	I don't feel like physical injury had impacted my interpreting, it's more mental health. I feel people who take care of their mental health can cope with difficult situations better. I often see people who exercise but while interpreting, they are stressed. From my point of view, the mind is more important than the physical **(Interpreter #27)**.<br><br>I see some interpreters... they seem stressed. They are worried if they are good or if the consumer accepts them and that mental stress influences their health. Mental stress can be destructive. Relax, let your mind go. I have learned through vocational rehabilitation counseling and training how to engage with people and consumers. Their situations can be awful… loss of a job, a change because of a new disability, the impact of a sickness… that's when my mind can

	when I was a staff interpreter, we did have that opportunity and that outlet to communicate and discuss. So, mentally, I think that is lacking here **(Interpreter #1)**.”<br><br>“I would love to see more focus in the future on mental health, as far as occupational health goes. I think interpreters have such a unique position and a unique job in that we are part of every part of people’s lives. So, I know for myself, I struggle a lot with the mental health part of interpreting. I have learned throughout the past 10 years that I don’t do as well with the really intense emotional assignments because I take that on and then, that affects me the rest of the day or depending on how horrible the situation is, maybe for the next week. So, I think I would love to see more focus on how to handle that or knowing that about yourself, doing self-analysis on, okay, I know that I can’t take those assignments but let me focus on more neutral assignments that I feel better with to reduce that stress **(Interpreter #1)**.”<br><br>“There’s an employee assistance program, but it’s very limited, like you can call up to this many times. You	level of anxiety, than I ever noticed before **(Interpreter #6)**.”<br><br><u>Individual strategies for managing mental health:</u><br>“As far as the corporate changes versus the individual changes, I appreciate, I do believe the corporation is trying to provide individual resources, I think they are, like the EAP counseling if you have a really stressful time; however, there are a lot of hoops to jump through if you want to get those resources, a lot. And, especially, if you are part-time.”<br>“When trying to go after those individual resources, what I end up doing anyway, is just finding ways that I can lessen my load because trying to advocate for carrying the load efficiently for me has not been working **(Interpreter #7)**.”<br><br>“...the thing that I have used is find a support system outside of the field of interpreting and outside of Deaf people. So, I have found it real important, I have a small group of friends/family members that if I	And, that’s my job some days. And I need to be able to come home and tell somebody about that who understands why I as someone who is supposed to be ethical and socially just (listing) and like meet all of these very high humanitarian mandates in my profession, had to make a choice between a bad choice and a worse choice today **(Interpreter #9)**.	breaks, there’s still always a million mental decisions and the emotional content, the psychological content, you know, you name it, the class content, the racial content of the work that we do that we carry with us and where does that go **(Interpreter #15)**?<br><br>I’m in the chair interpreting for a men’s group and they are talking about why it’s okay to beat women and they are talking about their experiences of, their intimate experiences with women and in that time, there were no men interpreters, there were none. So, I was interpreting for that. Somewhere inside of you, you have to be able to recognize what’s happening , recognize it’s not me, recognize that this too shall pass, and get done with the job with as much integrity in your interpretation as you possibly can and then you choose, do you go back and do it again or do you say you need to find somebody else? If you, as an interpreter, you’re in a situation that’s uncomfortable, 1) you have to do the job to the very best	attention to, you know **(Interpreter #17)**?<br><br>I wonder if you, once you find those other pieces of yourself if you’re more likely to advocate for your occupational health needs, you know, when you don’t have so much riding on this is my identity and who I am, I’ll die on the alter because that’s what I’m doing and that’s who I am, maybe it makes it a little bit easier to take care of yourself **(Interpreter #20)**.<br><br>I’m not saying it’s just the job, it’s one’s tendencies, it’s what you bring to it. Other people are not going to have this experience of not being able to be one’s self at, express your full selfness in the work, that’s what I felt, I wasn’t able to express being myself, who I am and I had to turn it off and that’s when I was, like… that was on like… I would say that was in the middle of burnout **(Interpreter #18)**.<br><br>I think that other thing too is that it’s important to recognize that a job may be super stressful for me or a client or a team. There are other interpreters who are highly skilled to do that and can be put in that position so the opposite of the totally oblivious interpreter who does not recognize their lack of skills, you have the highly skilled interpreter that you can put in the same situation that would put my hair on fire and it has absolutely not impact on them at all. So, I have	respond with no, but I could do this, like if it doesn’t work at that time, I’ll give them other times. But we were taught not to do that because we don’t want the interpreter’s schedule to dictate that Deaf person’s schedule of what they need or the hearing person’s availability. We were taught to deprioritize ourselves and to cram stuff in and then, it’s sortof a no-win situation because if I… okay, I have this other job until 2p, you want me at 2p? Is everybody okay with me, I have to leave right at 2p? And I’m going to be, like… and then, you get why aren’t you getting here 10 minutes early? What can’t you stay 10 minutes past? So, I just started to offer up options. For whatever reason in our field, we were taught not to do that but the clients seem very receptive to that **(Interpreter #21)**.<br><br>If I get a phone call from the emergency room and there’s someone who’s been in a bad accident, I can’t say, sorry, I don’t feel like coming. I will go. I will go.	have ELK **(Interpreter #25)**.<br><br>It’s important, upon receiving a job request, to read about the job before accepting it. Some interpreters just accept, responding quickly that yes, I’m available and go ahead because they need the money without realizing that when they report to the assignment they don’t know anything about what’s going on and subsequently, set-up the risk to themselves **(Interpreter #24)**.	be stressed. Through training and feeding my mind, I can manage, communicate better, and teach better health **(Interpreter #26)**…<br><br>During graduate school for rehabilitation counseling, I remember during my internship, I went to work and saw some terrible things and became emotionally involved. The teacher made a point to me saying, you cannot become emotionally involved with every case or you will become burned out. And that was 45 years ago but really stuck with me. I learned to protect myself. Luckily, I have many hobbies that also serve as my therapy and my escape. I have many outlets. If something sticks to me while at work, when I arrive home, I’m able to let it go before going into the house. I had children that I need to take care of. So, I learned how to cope and not let things from work stay with me. I have several positive outlets that help to fill me up when my tank is low. If something becomes a problem at work, I feel sorry

	can't guarantee that you're going to talk to the same person and to be perfectly frank, I don't know that any of us really trusts that what we are really talking about with whomever we are talking to, like how is this being reported to the district? Is it being reported that we did use it? Is it being reported that, hey, you need… like, I don't know that my rights under HIPAA apply to employee assistance. Cause, I don't know who these people are. I certainly didn't get anything from them in writing saying here are your privacy rights. Even though that benefit is there for us, nobody uses it **(Interpreter #3).**"<br><br>"I have thought about trying to go through the process of becoming a supervision leader because I see that need for it, but I also don't know how receptive colleagues and others might be **(Interpreter #3).**"	am either going some place that I feel very unsafe. I live in the South, I'm black. There are times that I am sent to places that it really is not safe for me and I'll go cause I'm not scared, but I will alert other people." "I do that and that gives me internal peace that I am just not out there in the wild world without anybody else being aware. Somebody needs to be aware **(Interpreter #6)**."<br><br>"The other thing that I have done and just to circle back to **<u>this idea of truth telling</u>** is I tell myself the truth about the state of affairs in this field. And I have intentionally been an observer of trends and allow the evidence to guide me where it goes versus (46:40) thinking about what ideally should be. So, I've dropped the word "should" out of my vocabulary **(Interpreter #6)**."<br><br>"...we have licensure and I have kept abreast of some, I'm gonna be kind, unfortunate short sided decisions that the licensure board has made and I actively engage in sending letters to say, hey, you need to think about the		of your ability, you cannot have an opinion and you cannot think it's distasteful, and you cannot think it's bad you have to become the person who is relishing in the pain and then when you leave you have to be able to say, but that's not me. You know? Mine, it comes in, it's dirty but it's not me **(Interpreter #13)**.<br><br>The work we do is an embodied work so it can't not have an impact **(Interpreter #14)**.<br><br>We do have control even where we think we don't have control **(Interpreter #14)**.	to… I try to go there despite my pessimism. I try to go they're going to get somebody who is a better fit for them than I am, someone who is qualified for this particular topic, situation, whatever the case may be, and recognize that I'm not the perfect interpreter for everybody. I have clients who like me, I have other clients who prefer to have someone else and I had to learn to be okay with that, too **(Interpreter #19)**.<br><br>That's interesting talking about not being able to be yourself because the one I've really enjoyed about what I'm doing in another professional realm is that I can be myself. I can say whatever I want whenever I want. I'm not sitting there relaying a message. I have a lot more space to just be who I am **(Interpreter #20)**.<br><br>It's like being able to use your own voice for your own self and I think that is important to be able to have something in your life where you're able to express your own voice **(Interpreter #18)**.<br><br>It's funny that you both mention that because in one of those "who am I?" workshops, they talked about that fact that I became an interpreter so that I could express, so that I could be the voice for other people, because I was not free to express my own, so all of my stuff was stuffed down, and I became an interpreter so I could be that voice for somebody else because I had lost my own voice **(Interpreter #19)**.	And the only way to keep myself with not having an occupational health problem is to say, you know what? Theater, you would like to have me come and interpret a play, I can't do that. Do you have Deaf people coming? Oh no, we just want access. Well, that's lovely and I've never done this in 25 years but I'm doing it now. Do you have Deaf attendees? Of course, we will make a way to do it. Do you not have Deaf attendees? It's wonderful that you want to provide access but I don't have the time or the people right now because in my world, that emergency room person is top priority **(Interpreter #22)**.<br><br>What I've done for myself in order to stay away from that feeling of overwhelm is I stay in the moment. I stay only in that moment. There's all this noise back here, there's all these people, and I had this happen a week and a half ago. I got a lady who is passing away in front of me. She's the most important person in		for that person, I give them my best for communication access, I work hard and make sure everything comes together for that consumer, but to bring it home with me, I don't do that **(Interpreter #27)**.<br><br>It takes training, in the beginning when you're young it's hard to manage, but with training, you can become more protective of yourself and carry on. To keep at a slight distance from this or that, you have to take care of yourself. I don't know if this applies to you [Interpreter #27] but for me, my relationship with God helps me so much. God helps me to make what happens smooth. I surrender over to God. It could be interacting with family, at work, and with friends, I use the same strategies to protect myself so that I can carry on. The training now has become engrained and I feel like I'm able to cope well **(Interpreter #26)**.<br><br>I know how to take care of myself. I work out. I go to the gym (54:16). I go and do things I really enjoy, it helps my mind detach. I

		ramifications of your decision, knowing it’s not going to go anywhere, but still, at least I know that they were informed. Those are ways so that I don’t get this feeling of helplessness because I firmly believe if I don’t understand how to garner, advocate, assess situations that put me at risk, there’s no way I can do that for Deaf clients **(Interpreter #6)**.”<br><br>“What I have found to protect myself because of the stress from interpreting VRS, is that I don’t go to the Deaf community much at all, which saddens me but at the same time it’s about the only way at this point I can protect myself from feeling so overwhelmed with that need to advocate at the same time I interpret and be a champion for the Deaf community **(Interpreter #7)**.”<br><br>“I do have several friends, in the interpreting community, that I can go to and debrief about that and say, errr, I just had this guy and I was trying to ask for clarification and he said bad interpreter (signed hang up). Hang up			I think that’s true for many and who is attracted to this field and the messages we’ve received about how to be a good interpreter. What it means to be a good interpreter can enable some of those innate qualities or mechanisms that worked when we were younger, you know, like what feels comfortable. It’s not to say that another interpreter has that same experience cause they come with a different kindof makeup but I think it does draw that **(Interpreter #18)**.<br><br>Until interpreters find their own voice, self-care will never be a part of their schema. Ever **(Interpreter #19)**.	my world at that moment and I don’t care about the rest. I’m staying in the moment for right here, right now with this lady, so the last thing she sees before her eyes close is someone signing **(Interpreter #22)**.<br><br>I have learned that when I stress out of those things that I used to stress at a younger age and go, oh, I don’t know how you do this. There’s become more of a quiet to it. If that makes sense? My experiences now are more quiet and they’re more, wow, did I really learn something from that. That’s an experience that I never want to forget because it was so beautiful. Whether it was a baby being born or someone passing and everything in between. And that doesn’t mean that we don’t cry and we don’t feel it. I get in my car and I cry all the time because I love the people I work with, but that inner peace that comes from being quiet over the situation because you were in the moment, changes everything. Instead of leaving and going, oh my goodness and I’ve		set rules for myself. I don’t bring problems to work **(Interpreter #27)**.<br><br>Sometimes hearing interpreters will share with me [their mental health problems] and I listen. If it goes on, sometimes I’ll give them tips. If they still don’t take my advice, then I am direct with them. I let them know that I don’t want to hear about it anymore because it seems you didn’t take my suggestions. I feel like I don’t want to continue talking about that because if I keep listening and I will take on some of your toxicity. You need to get professional help. I feel like I can’t offer any more, I establish boundaries **(Interpreter #27)**.

		on me. Why? You know? I think just putting it out there and “you’re still a great interpreter (signed double thumbs up).” Okay I’m good now. I’m good. Just having the comradery, that feedback, that support, whether it’s just (signed thumbs up), you’re doing a good job. Those things help me **(Interpreter #8)**.”<br><br>“Sometimes when work is getting really busy, the calls keep coming in super fast, you never know what type of call you’re getting, I’ll do something different. Like, puzzles online. Sometimes I’ll read a story to take me away from a place. That will help me to… something totally different… it helps your mind detach from that stuff **(Interpreter #8)**.”<br><br>When talking about enjoying the outdoors, biking, and taking up photography upon moving to Florida, “Getting out there and finding your passion outside of work, something that makes you feel alive **(Interpreter #8)**” was advice shared as a strategy for promoting her mental health.	There is this emotional component of just things that you’re not dealing with them but they’re coming through your hands and things that you experience, things that, like the vicarious trauma that happens when there is a lack of mentorship. Even if it’s not, I’m trying to become a certified interpreter and I need a mentor to help me get ready for the test, but just like I need a mentor to kindof to show me how you stay afloat through all of this **(Interpreter #12)**.			got to drive home and it’s crazy and I’m gonna get home and I’ve got all these things to do, I stay in that quietness and it kindof is healing, very healing **(Interpreter #22)**.<br><br>While you [Interpreter #22; about death and dying] were talking it made me think about the occupational hazard of like, your well-being. Your emotional well-being, like seeing those people go through that and like you’re saying, you’re prioritizing that person, you’re staying in the moment, you’re doing that, but what if you do that today, tomorrow, the next day, the next day, the next day and you never have the chance to kindof look up and take a breath, right? And then, that toll on you **(Interpreter #21)**...<br><br>One thing that I do is that I listen to Podcasts. So, if I’m onsite doing an interpreting assignment and I’m driving home, so I have that time between work and home… listen to a Podcast, totally different, change gears, hopefully it is something that is light or whatever.		

		"What I am experiencing at home is that the expectations of use of time are much stricter than they were when I was in the [VRS] center. Maybe those haven't technically changed but the perception of them has. So even Deaf clients are expecting that the interpreter is going to be doing this all the time (snap, snap, snap). So, I resent that, often and I find myself not wanting to follow the rules (smile, laughter) but I do, I'm a good interpreter. But, what I find, is that it's **really a stressor on my mental health**, where I have outside factors that are affecting me right now with caregiving and I had a broken leg for the fall – I mean, it was just a few things like that. I am finding that my mental health is also compounded with what I'm being expected to do at my job as an interpreter **(Interpreter #7)**."				And then I get home and I'm ready to come in. Before I did that, I'd come home and I'd be just like, you know, stressed and I'm, you know, that stacking thing. You've got 20 different things that kindof went hard that day and then something, little minor happens at home and you blow up and you're like, why? That wasn't even a big deal but I'm mad or I'm emotional and I don't even know why so that, with that destressing or decompression time is helpful **(Interpreter #23)**.<br><br>You have to schedule time for, like intentional decompression but we don't maybe realize that we have to do it and then, those, it just, the time slips away and you don't… when you're in the car it just sortof organically kindof gives you that opportunity to just like think through or listen to some music or listen to a podcast or just look at the world around you, you know? And, it's just, maybe that, like having to be intentional about it makes it easier to forget to do it or easier not to do it **(Interpreter #21)**.		

						When I have a particularly hard week, like last week, I turn to my faith. I listen to uplifting message music in the car and I decompress that way **(Interpreter #22)**.<br><br>Where ever interpreters can have more autonomy, I think would be helpful because there’s so much time that we spend where we don’t have a lot of presence as just who we are so I think kindof overlapping with that professional identity, so as far as a community of practice, for example, or even like a training, right? Is like having that ability to, to have some autonomy and some self-selection with that **(Interpreter #21)**.		
