# Supplementary material for: Occupational Safety, Health, and Well-Being Concerns and Solutions for Management Reported by Sign Language Interpreters: A Qualitative Study
Source: Int J Environ Res Public Health. 2024 Oct 23;21(11):1400. doi: 10.3390/ijerph21111400 (PMC11594195; doi:10.3390/ijerph21111400)
Supplement: Supplementary file 1 [file ijerph-21-01400-s001.zip › ijerph-3194280 supplementary/ijerph-3194280-supplementary.docx]

| **Table S1.** Standards for Reporting Qualitative Research (SRQR) [41] | |
| --- | --- |
| **Title and abstract** | **Page/line number(s)** |
| **Title** – Concise description of the nature and topic of the study. Identifying the study as qualitative or indicating the approach (e.g., ethnography, grounded theory) or data collection methods (e.g., interview, focus groups) is recommended | Page 1, lines 1-4 |
| **Abstract** – Summary of key elements of the study using the abstract format of the intended publication; typically included background, purpose, methods, results, and conclusions | Page 1, lines 15-30 |
| **Introduction** | **Page/line number(s)** |
| **Problem formulation –** Description and significance of the problem/phenomenon studies; review of relevant theory and empirical work; problem statement | Pages 1-3, lines 35-115 |
| **Purpose or research question** – Purpose of the study and specific objectives or questions | Page 3, lines 115-118 |
| **Methods** | **Page/line number(s)** |
| **Qualitative approach and research paradigm** – Qualitative approach (e.g., ethnography, grounded theory, case study, phenomenology, narrative research) and guiding theory if appropriate; identifying the research paradigm (e.g., postpositivist, constructivist/interpretivist) is also recommended; rationale** | Pages 4, lines 162-176 |
| **Researcher characteristics and reflexivity** – Researchers’ characteristics that may influence the research, including personal attributes, qualifications/experience, relationship with participants, assumptions, and/or presuppositions; potential or actual interaction between researchers’ characteristics and the research questions, approach, methods, results, and/or transferability | Pages 3-4, lines 149-151 |
| **Context** – Setting/site and salient contextual factors; rationale** | Page 4, lines 154-155 |
| **Sampling strategy** – How and why research participants, documents, or events were selected; criteria for deciding when no further sampling was necessary (e.g., sampling saturation); rationale** | Page 3, lines 126-144, Page 4, lines 175-176 |
| **Ethical issues pertaining to human subjects** – Documentation of approval by an appropriate ethics review board and participant consent, or explanation for lack thereof; other confidentiality and data security issues | Page 3, lines 120-124; Page 21, lines 580-585 |
| **Data collection methods** – Types of data collected; details of data collection procedures including (as appropriate) start and stop dates of data collection and analysis, iterative process, triangulation of sources/methods, and modification of procedures in response to evolving study findings; rationale** | Pages 3-4, lines 145-161 |
| **Data collection instruments and technologies** – Description of instruments (e.g., interview guides, questionnaires) and devices (e.g., audio recorders) used for data collection; if/how the instruments(s) changed over the course of the study | Page 4, lines 154-155 |
| **Units of study** – Number and relevant characteristics of participants, documents, or events included in the study; level of participation (could be reported in results) | Table 1 |
| **Data processing** – Methods for processing data prior to and during analysis, including transcription, data entry, data management and security, verification of data integrity, data coding, and anonymization/de-identification of excerpts | Page 4, lines 164-167 |
| **Data analysis** – Process by which inferences, themes, etc., were identified and developed, including the researchers involved in data analysis; usually references a specific paradigm or approach; rationale** | Page 4, lines 168-176 |
| **Techniques to enhance trustworthiness** – Techniques to enhance trustworthiness and credibility of data analysis (e.g., member checking, audit trail, triangulation); rationale** | Page 4, lines 172-176 |
| **Results/findings** | **Page/line number(s)** |
| **Synthesis and interpretation** – Main findings (e.g., interpretations, inferences, and themes); might include development of a theory or model, or integration with prior research or theory | Pages 5-10, lines 198-405 |
| **Links to empirical data** – Evidence (e.g., quotes, field notes, text excerpts, photographs) to substantiate analytic findings | Figure 1, Supplementary materials: Table S2, and Table 2 |
| **Discussion** | **Page/line number(s)** |
| **Integration with prior work, implications, transferability, and contributions to the field** – Short summary of main findings; explanation of how findings and conclusions connect to, support, elaborate on, or challenge conclusions of earlier scholarship; discussion of scope of application/generalizability identification of unique contributions(s) to scholarship in a discipline or field | Pages 18-21, lines 406-554 |
| **Limitations** – Trustworthiness and limitations of findings | Page 20, lines 543-554 |
| **Other** | **Page/line number(s)** |
| **Conflicts of interest** – Potential sources of influence or perceived influence on study conduct and conclusions; how these were managed | Page 21, lines 593-594 |
| **Funding** – Sources of funding and other support; role of funders in data collection, interpretation, and reporting | Page 21, lines 577-581 |
| *The authors created the SRQR by searching the literature to identify guidelines, reporting standards, and critical appraisal criteria for qualitative research; reviewing the reference lists of retrieved sources; and contacting experts to gain feedback. The SRQR aims to improve the transparency of all aspects of qualitative research by providing clear standards for reporting qualitative research. | |
| **The rationale should briefly discuss the justification for choosing that theory, approach, method, or technique rather than other options available, the assumptions and limitations implicit in those choices, and how those choices influence study conclusions and transferability. As appropriate, the rationale for several items might be discussed together. | |

| **Table S2.** Occupational safety, health, and well-being concerns of sign language interpreters | | |
| --- | --- | --- |
| Domain | Exemplar quote | |
| Mental health | [regarding the new set of interpreting demands since COVID] As human beings, I’m noticing interpreters, my own colleagues, are far more afraid than they were in the past. I’ve noticed there’s a higher level of anxiety, than I ever noticed before (Interpreter #6). | |
|  | When people get over that [the physical part or mechanics of interpreting] or perhaps when they work in situations where they have teams and the work environment is less demanding and more forgiving in terms of time and breaks, there’s still always a million mental decisions and the emotional content, the psychological content, you know, you name it, the class content, the racial content of the work that we do that we carry with us and where does that go (Interpreter #15)? | |
|  | If we are not taking care of ourselves, it makes it even more stressful. People laughed at me when I was looking for some way to balance out the physical/mental/emotional stress of the work. And I think it’s so important. What I’m hearing so much is that people don’t have, even getting up and just taking a walk, like moving, as simple as that is, people are not finding the time to do that. I do think people are doing other sorts of unhealthy things to make it through and so that’s going to impact the occupation (Interpreter #18). | |
|  | Race and disability can impact the variable. BIPOC interpreters talk about how they feel more stress that impacts their overall health. Deaf interpreters, as well, feel stress. We need to do follow-up research to focus on BIPOC and deaf interpreters. I’m sure it is a lot higher than white interpreters (Interpreter #25). The same for LGBTQ interpreters, rainbow interpreters, as well (Interpreter #24). | |
|  | I see some interpreters... they seem stressed. They are worried if they are good or if the consumer accepts them and that mental stress influences their health. Mental stress can be destructive (Interpreter #26). | |
|  | I don’t feel like physical injury has impacted my interpreting, it’s more mental health. I feel people who take care of their mental health can cope with difficult situations better. I often see people who exercise but while interpreting, they are stressed. From my point of view, the mind is more important than the physical (Interpreter #27). | |
|  | Sub-domain | Exemplar quote |
|  | Workplace violence | I surveyed about 10 behaviors [e.g., scapegoating, breaking of confidences, gossip] that are associated with horizontal violence. And found that for 7 of those 10 behaviors, a majority of respondents said that they had either experienced those things themselves from other interpreters or from Deaf community members or they had witnessed those things happening in their community (Interpreter #9). |
|  |  | A lot of the literature that I was reading pointed to any identity that is divergent from the hegemonic culture being a target. That bore out in my own experience. Upon presenting to a person who will identify me as white, fem, neurotypical, they will interact with me in one way and then, when I self-disclose my identities, they will interact with me in a different way (Interpreter #9). |
|  |  | Experienced, seasoned interpreters in my community were very resistant to new interpreters. I couldn’t find a mentor. That began a very long gap for me between graduation and getting any kind of credential and then, getting some kind of credential to finding work. That 5-6 year period included a lot of established professionals in my community being harmful to me, either outright aggressive or passively aggressive… bullying (Interpreter #9). |
|  |  | Agency violence is the prominent and most harmful source of interpreter fatigue, burnout, pain, disaffection, writ large. There is no benevolent agency. When I started working for myself, everything changed. I try to help other people, who report similar experiences, do the same thing (Interpreter #10). |
|  |  | I’ve thought about the horizontal violence in our field. There are messages between interpreters that being selective about the work is not professional. I don’t like to hear people say, “I don’t do medical,” “I don’t do legal,” “I don’t do…” whatever, or like, how can you not work with that team interpreter when you know that that Deaf person needs coverage? We do it to ourselves and we do it to each other (Interpreter #21). |
|  |  | If I team with a hearing interpreter, in theory from a professional perspective, the two of us are horizontal. But, when you think about the system of power, it is not equitable, so it becomes vertical violence (Interpreter #25). |
|  |  | Data from one of my studies back in 2016, it wasn’t part of my focus, but a Deaf interpreter said they felt that Deaf interpreters bully each other more than hearing interpreters when it relates to ethical decisions (Interpreter #25). |
|  |  | [regarding why Deaf interpreters tend to bully each other more] Because of the limited ITP, because of the limited training… when Deaf interpreters get together, there are different approaches. A Deaf person’s upbringing, being deflated by a hearing teacher saying, “that’s not right.” It’s hard for Deaf interpreters to separate from the oppression they experienced growing up upon entering into the interpreting profession. Sometimes, it’s an uneasy feeling when you see a Deaf interpreter being so critical. How to be tactful? “I’m curious about your approach?” “Why did you decide to pick that sign?” Instead of, “You signed that wrong, you’re stupid to use that!” in front of the client (Interpreter #24). |
|  | Secondary traumatic stress or vicarious trauma | Somewhere inside of you, you have to be able to recognize what’s happening, recognize it’s not me, recognize that this too shall pass, and get done with the job with as much integrity in your interpretation as you possibly can and then you choose, do you go back and do it again or do you say you need to find somebody else? If you’re in a situation that’s uncomfortable, 1) you have to do the job to the very best of your ability, you cannot have an opinion and you cannot think it’s distasteful you have to become the person who is relishing in the pain and then when you leave you have to be able to say, but that’s not me. It comes in, it’s dirty but it’s not me (Interpreter #13). |
|  |  | The work we do is an embodied work so it can’t NOT have an impact (Interpreter #14). |
|  |  | I was going to talk a little bit about designated interpreting because that’s an interesting blessing and curse, I have found. It does this wonderful thing where you have relatedness and consistency of service for that Deaf person and your own knowledge base building around how they want to be represented. I LOVE working with the same Deaf person over and over again and guess what? Anything they go through will impact you. I mean boundaries are important, like I’m not going to go save the Deaf person or fix their problem, but the whole vicarious trauma thing. I think people think it’s AN incident where to me, not to be overly dramatic, it’s like death by a thousand cuts. So, you’re taking it and taking it… you’ve GOT TO have something that counters all of that (Interpreter #16). |
|  |  | [talking about death and dying and emotional well-being] Seeing people go through that and you’re prioritizing that person, you’re staying in the moment, but what if you do that today, tomorrow, the next day, and you never have the chance to look up and take a breath? And then, that toll on you (Interpreter #21)... |
|  | Lack of work-life integration or boundaries | What’s missing? When interpreting is too overwhelming, the pieces that are missing are the pieces of yourself that you’re not giving attention to (Interpreter #17). |
|  |  | I wonder if, once you find those other pieces of yourself, when you don’t have so much riding on this is my identity and who I am, if you’re more likely to advocate for your occupational health needs (Interpreter #20). |
|  |  | There are comments from within our community about the folks who are trying to set boundaries. “Well, people don’t want to work” or “they’re very selfish.” I think that it’s healthy to do that. I think that it’s difficult to do that. I think it’s easier to have boundaries first, than to have not had any boundaries and then, try and establish some. We have that internal self-talk, but you don’t get positive reinforcement from colleagues or consumers that boundary setting is healthy or appropriate (Interpreter #21). |
|  |  | It’s just impossible [to take a day off] and that affects us as interpreters, that’s part of occupational health. You can’t continue to work 60 hours a week for 52 weeks as much as we want to because I always look at it as, who am I going to say no to? Who gets to sit there without an interpreter? Which one is more important? They’re both important! I want to be both places but I can’t (Interpreter #22). |
|  |  | During COVID, you couldn’t get in to see a medical provider, you couldn’t get into the see a specialty vet because they were limiting their work and still a lot of them left [their fields]. Now, it’s like 2-3 months to get into an appointment and you’re like, “yea, but I’m suffering now.” They’re like, “our next available appointment is in 3 months.” I wish we [the interpreting field] could take something from that because they’re definitely using self-care. They are saying, I have 8 hours to my day, I have this many medical slots for appointments and the next available slot is in 3 months, “do you what it?” And we’re like, “okay, so maybe I can fit you in.” We’re not there, but maybe we might learn something (Interpreter #23). |
|  | Loss of agency or loss of self | I’m not saying it’s just the job, it’s one’s tendencies. It’s what you bring to it. Other people are not going to have this experience of not being able to be oneself, express your full selfness in the work, that’s what I felt. I wasn’t able to express being myself, who I am and I had to turn it off. I would say that was in the middle of burnout (Interpreter #18). |
|  |  | I became an interpreter so that I could express, so that I could be the voice for other people, because I was not free to express my own. All of my stuff was stuffed down and I became an interpreter so I could be that voice for somebody else because I had lost my own voice (Interpreter #19). |
|  |  | Until interpreters find their own voice, self-care will never be a part of their schema. Ever (Interpreter #19). |
|  | Isolation | I go in these waves where I’m really good, I’m feeling like a team, if we can see each other or we get together… and then, sometimes, I feel super isolated. I have no idea what anybody else is doing. It doesn’t feel good (Interpreter #4). |
|  | Setting-specific | I have chosen to not do VRI or VRS work because, to me, it is super stressful. I’ve heard stories. The demands that are put on you. I want to be able to control what I’m walking in to. During COVID, we were virtual for school. That was just so stressful for me. We’re here to provide a service and we do it because we love what we do. I never came into this profession thinking, “oh, I’m going to make so much money.” I’m not gonna put myself out there to just make more money. My mental health is more important than that (Interpreter #5). |
|  |  | [inability to request a remote team for 9-1-1 calls because the Deaf person’s view of the interpreter becomes too small] ... for that reason, I choose not to team and sometimes it’s at my own expense (Interpreter #8). |
|  |  | [during a suicidal mental health call, technology allowed the Deaf person to see the interpreter and the hearing provider but limited the interpreter from requesting a remote team] I could have really used a team. It was a very traumatic situation but I stuck with it for the benefit of communication. That’s really tough and then, you only have like a 10-minute break. You’ve got to brush it all off and be ready, okay next call (Interpreter #8). |
|  |  | Part of what contributes to the mental health of freelance interpreters and their longevity in the field is understanding that it’s not 9a-5p, it’s not a job where you have a boss who tells you what to do. You have choice to take a break when you need to without fear of losing your job (Interpreter #13). |
|  | Limited extra-linguistic knowledge (ELK) | Interpreters with limited ELK [don’t know the topic] seem more likely to stay with the form [instead of interpreting across two languages, interpreting within the same language]. I see more stress and processing while signing because they don’t understand the information. They don’t feel connected with their whole body. They say that they know the topic, that it’s easy, that they can use their prediction skills, that they can process, but it’s more of the same form. They fingerspell more, so that can have an impact. Compared to the interpreter who knows that topic, more free form, and can figure out how to sign. Based on my observation, I wouldn’t be surprised if interpreters who have ELK hurt less (Interpreter #25). |
|  | Exemplar quote | |
| Organizational culture | Because of the way that our industry is and because of the system that we operate within, we’re forced to make choices between a bad choice or worse choice and that’s my job some days (Interpreter #9). | |
|  | I dream of a world where interpreter preparation programs give a more fulsome level set of expectations, business practices… the kinds of things that we’re talking about… rather than just do this before every job [modeling wrist stretches]. Actually protect yourself, like in a real way (Interpreter #10). | |
|  | Yeah, there are definitely occupational hazards but it’s like, either from within, it’s my fault, the way I hold my body, the way I sign or it’s from within the culture and the way that we can eat our young or not support each other (Interpreter #12). | |
|  | It’s not in our culture that this [occupational health] is important, like that you’re an athlete or a dancer or somebody that uses their body. You must train and watch your sleep, your nutrition, everything, your mental health… this is not at all part of our work (Interpreter #18). | |
|  | [after call volume spiked during COVID] The [VRS provider] company started to say more, “okay, take your breaks.” But people wouldn’t. We have this group and we message each other and would be like, “oh, I’m really sorry, I just need to take a quick break. I have to go get a coffee.” And I’m like, why are you telling me that? Just go do it. Why do you feel like you need to apologize for taking care of yourself? Even when the supervisors were saying, “hey, just do whatever you need to do.” People were still almost like, very apologetic for needing to do that (Interpreter #20). | |
|  | As hearing people, we’re taught that we need to be weary of our oppressive behaviors. I’m a hearing person, so I shouldn’t complain about my aches and pains. I shouldn’t complain about my busy schedule. I should just keep going in service of because Deaf people go without all of these things, all of the time. If I have to sleep a little less or have to do such and such… that rhetoric, I think, gets pervasive in our field (Interpreter #21). | |
|  | Sub-domain | Exemplar quote |
|  | Deprioritization of self | I do wonder how much you’re trained to deprioritize yourself and so, if we do have that training or perhaps that tendency... it was really difficult for me to come to a point where I was like, actually, I can’t. I can’t cope with this (Interpreter #20). |
|  |  | …self-worth, like viewing yourself as a person who is worthy of care and love that is not just this machine that is here to serve and take care of other people (Interpreter #21). |
|  |  | I also think there’s a cultural component. We’re taught to deprioritize ourselves, our own well-being. I know that I should drink more water, take breaks, lower my arms, and do all of these things. I wake up in the morning with the plan to do that and then, it’s 11p at night and I haven’t done it. More so than there being any one particular job that had some negative impact on me, it’s more cumulative (Interpreter #21). |
|  |  | We are so service oriented. I know I am. I’m thinking of the person [Deaf consumer] that is there. We have the servant’s heart, that’s what we do, we serve people. I can’t turn that off (Interpreter #22). |
|  | System or organizational challenges | As someone who works as a staff interpreter in a school district, our HR department is not there for us as employees. Our HR department is there to protect the district in any situation that involves employees or involves consumers of the educational services that our district provides. That means we are left fending for ourselves, if we have concerns about our occupational safety and health. The demands that have been put on us as far as doing more and feeling like they just don’t care. As long as they are able to check the box that the IEP is in compliance, they don’t care (Interpreter #3). |
|  |  | I have worked in some organizations and I’ve found that the only push for occupational health came from me. Like pushing at them to provide intentional breaks for workers, provide intentional access to physical fitness exercises, to mind breaks, but I felt like I’ve never had that pushed on me, it was always me pushing at them (Interpreter #17). |
|  |  | You have your individual and then, you have your organization. I’m thinking of organizations, like agencies or RID or state chapters and then, the larger systems… it starts to feel overwhelming. Even if I do Crossfit and I do time management and I get a good night’s sleep and I try to bring more interpreters into the field, like there’s still going to be something about this work that is going to be hazardous to the health of the people that are doing it (Interpreter #21). |
|  |  | Some interpreters have more basic health insurance because that’s all they can afford in the marketplace. When they are asked what caused it, they say their job and that insurance doesn’t cover it, so that becomes a problem for interpreters (Interpreter #25). |
|  | Oppression | The vicarious trauma of knowing and seeing that the system is not set-up for Deaf people… if a police department doesn’t think to call an interpreter or if no interpreter is available or if I decide that I need to prioritize sleep because tomorrow I’m interpreting for something very high stakes, like the systems that we’re working within... Not even if there were more interpreters available, but if people working at the intake desk in the police station knew ASL and they could communicate directly with that Deaf person… go through to check the boxes that they needed to, then the interpreter shortage wouldn’t be a problem, right? Those things that are way out of the control of what we can do as interpreters but that we see every single day, I think contribute to the strain of the job because that’s part of why we feel like we can’t say no because we know there’s no other option (Interpreter #21). |
|  |  | I decided to do a survey of Deaf interpreters about their experience working with hearing interpreters and hearing agencies. 100% of the participants said that they still experience oppression on the job from the hearing team and around 60-something percent experience agency oppression (Interpreter #25). |
|  |  | Now, power and privilege sessions are about white oppression of people of color. There is a place and time where we have to address interpreters’ attitudes. As a Deaf professional, I am still looked down upon. I remember when I first moved to the area, I went to my first workshop and several hearing interpreters asked me, “did you graduate from an interpreter training program?” I looked at them thinking, “wow.” I responded by saying, “I don’t need an interpreter training program.” They asked, “why not?” I have 4 college degrees and still that wasn’t good enough for them (Interpreter #27). |
|  | “isms” | At the first Deaf interpreter conference, we discussed whether Deaf interpreters should be Deaf from Deaf families. Because Deaf-of-Deaf have more language fluency, are more from Deaf institutes, and have more of the Deaf experience and a stronger use of ASL. I disagree… I am Deaf-of-Deaf but I feel that Deaf-of-hearing have a lot to bring, as well. Their Deaf experience and their language matches some Deaf people. For example, if a Deaf person grew up oral, maybe I wouldn’t be a good fit because I wouldn’t have that understanding. So that is called… I’m not sure what “ism” (Interpreter #25). Elitism (Interpreter #24)? |
|  |  | The second one [“ism”] is not discussed much, sexism. When I went to the first Deaf interpreter conference, there were more men. The professional interpreting field was established in the 1960s. It was a helping profession. Interpreters are helpers, like social workers and teachers. They were not of elevated status and the pay, back then, was lousy. This perpetuated more women in the field because women have a heart for the work and are more compassionate. Now, as the interpreting field has become more elevated with better pay and better opportunities, and so on… men tend to take control, so I’m seeing that. Another example of sexism… most certified International Sign Language interpreters by the World Federation of the Deaf are white men. The was a hot debate at the World Association of Sign Language Interpreters conference in Paris 4 years ago. Why are there not more females (Interpreter #25)? BIPOC (Interpreter #24)? If you look during COVID, who interpreted the most on television? Men. I used to work at [name of an academic medical center]. I remember 5 Deaf male interpreters controlled the job opportunities at that hospital. They would interpret labor and delivery, rape violence, and some Deaf women would call me crying and asking, “why weren’t you available?” And I would respond, “I have been available and ready to work but they [Deaf male interpreters] control that.” I’m seeing the same thing with [VRS provider]. They have many male Deaf interpreters. I have asked them, “why do you interpret labor and delivery, rape, gynecological appointments?” And they say, “I’m just a professional interpreter. I’m neutral.” Research already proves that it’s impossible to be neutral. So, sexism, is something we need to look at (Interpreter #25). There is not only the binary system, male and female, but also non-binary. This is difficult for LGBTQ interpreters (Interpreter #24). |
|  | Relationships with interpreter peers and Deaf community members | What is your relationship with other interpreters, how do you define a healthy relationship? How do you define a healthy relationship between you and Deaf people regardless of setting? How do you develop a healthy relationship between you and customers and outside of that, your family… we never talk about our relationships and how that needs to happen (Interpreter #6). |
|  |  | One of the things I feel like it’s very much about health and longevity in the field is relationship to Deaf community and relationship to peers. Everybody knows everyone. When the Deaf people say, “my son has to go to therapy, can you please come?” Even if I don’t really want to. When Deaf people ask you and you really connect with them that’s both support and pressure. It’s hard to say no. It’s hard to say, I need to do this for my mental health when in fact their mental health in on the line. How does that support us staying in the field and how does that pull more from us than we have to give? And what are our relationships to our interpreter peers? How are we supporting them and how are they supporting us? I think a lot of people will suffer from having been criticized from peers and teachers and feel nervous… I think it takes some maturity or desperation to actually say, “I’m struggling here, I need some help (Interpreter #15).” |
|  | Not being seen as equivalent professionals | I think there is a lack of education for people who we work with, especially in a medical field, that don’t see us as equivalent professionals. I think there’s needs to be an education component that, “hey, we have occupational health concerns, as well (Interpreter #1).” |
|  | Exemplar quote | |
| Physical health | The physical stuff, you have to sign in unnatural ways sometimes, and this can cause problems in your limbs and maybe other places. Also, I have found that eye strain and eye spasms are real if I don’t take care to do that right. In Europe, among spoken language interpreters, they have revolted due to hearing loss problems. Because of the way volume goes in and out, sometimes you’ll get a blaring feed of a terrible scratchy mic and sometimes it’s very faint so you have to crank up your volume. It’s not leveled and it’s not occupationally safe (Interpreter #10). | |
|  | I think that the emphasis on ergonomics and signing, is hurting interpreters and it just makes me sad and a little bit frustrated (Interpreter #13). | |
|  | So as a brand-new interpreter, I got a repetitive motion injury and it was very obvious why, I was working with four Deaf professionals, 175 Deaf clients, and I barely had passed the state Q&A exam. It was a 6-8 month period of time when I had the injury and it was so about being an L2 learner and doing way too many hours of interpreting. I was doing 60-hour weeks, so it wasn’t too surprising that that happened (Interpreter #16). | |
|  | To earn the money I needed, I had to do something different. Physically, I had to do something different cause I wouldn’t have been able to sit here in this chair for all the hours I needed or anywhere, even face to face, I couldn’t do it anymore (Interpreter #20). | |
|  | I would like to have the recent grads and the newer folks to not have to be pushing their bodies so hard that they’re potentially driving themselves out of the field or creating more of the aches and pains. You just kindof deal with it, push through it, get ice packs, and go to physical therapy and stuff, you know? If we could avoid that all together, I think that would be great (Interpreter #21). | |
|  | Regarding ergonomics, I’m fascinated by how interpreters sit while signing; in particular, hearing interpreters. When fingerspelling, there is tension [demonstrates fingerspelling with tension using the right hand and relaxed using the left hand]. I’m concerned because I’m noticing more and more younger interpreters getting hurt earlier. I ask them, “why do you do that?” They say they are tense because they want to be clear. Well, you can be clear without that tension (Interpreter #25). | |
|  | Sub-domain | Exemplar quote |
|  | Physical effects of non-physical work (emotional, cognitive, and linguistic) | [upon conducting onsite evaluations with independent contract or staff interpreters] One of the aspects that I find myself having to focus on is avoidance of injury and how just to posture, not even how your hands flay around but the notion of sitting up straight, keeping your shoulders down and tuning into what your body is doing under any circumstance because interpreting can be stressful. Even before you get started, if you know what to look for, you can scan somebody’s body and immediately see, yep, there’s something, they’re stressed about before they get started (Interpreter #6). |
|  |  | We’re talking about the mechanics of sign production, which is only really one part of interpreting [spoken English to ASL]. I’m wondering about going from ASL to spoken English, which is part of our work that interpreters don’t do a lot or shy away from and I think is also tension producing (Interpreter #15). |
|  |  | If you look at spoken language interpreter training programs, they tend to require that students already be fluent in that second language. But for sign language, we start teaching language for two years and then, they go on to interpreting. It sounds like that is an open opportunity for increased injury because they are not comfortable using that language (Interpreter #25). They are set-up for failure (Interpreter #24). Why do I say that? Do I see Deaf interpreters hurt? Not really. If I get done interpreting all day, not Deaf-Blind interpreting, but interpreting all day with a team, switching back and forth, I am fine. My brain is tired from the processing, but my body is okay. I see interpreters [demonstrating signing awkwardly with tension]. They were not comfortable with the language to begin with when entering into the field of interpreting (Interpreter #25). |
|  | Aging | I am really interested in, what does it look like for an interpreter moving in to their 50s and 60s and how you sustain that in a healthy way (Interpreter #2). |
|  |  | When I think back to when I was first starting out and much younger, how I carried my body, how I would sit, like what I could tolerate physically was so much different than what I can tolerate now (Interpreter #3). |
|  |  | I’ve had to take more care as I’m getting older with certain physical things that I’m noticing. It’s a big, everything-is-related type of situation. But I’ve never gone very deep with understanding how these factors affect me because I prided myself in being the person who is athletic and healthy and doesn’t worry about it until I’m 69 years old now and it’s not a given anymore (Interpreter #11). |
|  | Setting-specific | I am noticing that, physically, my body is hurting more. In person, there’s a meeting and then, physically you have to walk to a different place. So, a minute, two minutes where you can breathe and get some rest. Whereas here [VRI], it’s like okay [signed a window popping up on the computer], go [signed interpret], and then, you’re done, next class! You don’t have time to breathe, drink water, or go to the bathroom (Interpreter #4). |
|  |  | I work with Deaf-Blind consumers. In the beginning, I was uneasy. I went to a training and practiced and felt some pain. I knew from my experience in the corporate world that I should stop if something was causing me pain. But how can I continue working with the Deaf-Blind and figure out some balance? That forced me to practice with my left [non-dominant] hand to save my right and balance out the burden. Now, I’m skilled with both hands while signing. That doesn’t mean that I can interpret for a long time alone. Always, when I get called for Deaf-Blind interpreting, if I have a team and we can switch off with one another, that is ideal. But if not, I always let them know if I am the only interpreter, we have to figure out my constraints. The availability of Deaf-Blind interpreters is very limited. Many have left because they hurt themselves (Interpreter #24). |
|  |  | I have been hurt on the job. I was interpreting for someone who is Deaf-Blind. They were new to tactile signing and heavy on my hand. That was a lot on my shoulders. I had to go to physical therapy. I thought, I’ll be fine after physical therapy helped me to improve. I remember when I went back and did an immersion week for protactile interpreting. For that week, my right shoulder hurt again. I decided not to do protactile interpreting and I miss it. I do feel connected with the Deaf-Blind community, but I feel maybe other interpreters have healthier shoulders than I do (Interpreter #25). |
|  |  | Deaf-Blind consumers were upset and wanted me to still interpret but I had to say, I’m sorry because of how it affected my shoulders. Those interpreters for Deaf-Blind consumers have to be taught how to exercise to help them get back to Deaf-Blind interpreting. Maybe that will change their health, but right now I cannot do it because of how it impacted my shoulders (Interpreter #26). |
|  | Exemplar quote | |
| Difficulty dialoguing about the work (debriefing), describing rationale for ethical decisions, and offering/receiving feedback | We do not know how to talk about our decisions. I often see interpreters bully against each other over ethical decisions that they don’t feel they align with. It’s really simple, why not ask, “I’m curious why did you make that decision?” To understand each other’s perspectives. “Well, I would have chosen…” That conversation almost never happens in the interpreting field. Why are you bullying the interpreter? Did you ask them why they made that decision? You just saw the decision and you got upset but you don’t know the whole story, why that interpreter decided to do it that way. I would decide that way, why? And then, discuss. That give and take or exchange of information is needed but that is not happening, so really it increases the horizontal violence because we don’t know how to talk with each other (Interpreter #25). | |
|  | Hearing interpreters can be very orderly in their interpreting. I mix up the message to match the Deaf consumer. Yes, conflicts do arise in our discussions that bother me and them but we have to work together to understand each other. I know the Deaf community needs us (Interpreter #26). | |
|  | I don’t always discuss the sign interpretation and say that word needs to improve but also, for example, in the medical field, one consumer/patient had one week left to live. That really stuck with me. The patient would say, “I’m healthy, I’m fine.” Upon checking with the doctor, we let him know that no, he was dying. He kept insisting that he was fine in effort to try and keep himself alive. But in the interpretation we conveyed, you will die, do you know what I mean? It really hit me, that consumer will die. After that, I needed to talk things over with my team, give each other a hug, then be on our way, it was healthy to do that. If not, then I wouldn’t know what to do with that. I want to add that that type of debriefing is really necessary (Interpreter #26). | |
|  | I work for [VRI provider]. They tell me I have to discuss after each VRI assignment. For the discussions, I don’t have that kind of training to know what kindof feedback to provide. After some interpreting situations, I go up to the other interpreter talk directly with them and discuss a matter of things, particularly if there was something I didn’t agree with. Some interpreters are able to receive that well, other interpreters cannot handle it very well. I wonder if my feedback creates a benefit or separation (Interpreter #27). | |
|  | Sometimes hearing interpreters share their mental health problems on purpose because they don’t want feedback. After the job is done, they take off stating they have another job they have to get to. That kind of interpreter needs a lot of feedback. They know they are not that great but they are in it for the work and then they take off (Interpreter #27). | |
|  | I’m not sure if I trust the hearing interpreter for debriefing. When I’m direct with the hearing interpreter, I’ll say, “can’t you see the Deaf person’s body language? It looks like they don’t like you talking at a lower level to them.” When I see that it gives me pause. I treat the Deaf consumer equally with dignity. I know they may have limited education, a limited vocabulary but I never show that I’m treating them at a different level from me (Interpreter #27). | |
| Work-life interference | What I am experiencing at home is that the expectations of use of time are much stricter than they were when I was in the [VRS] center. Maybe those haven’t technically changed but the perception of them has. So even Deaf clients are expecting that the interpreter is going to be doing this all the time [signed snap, snap, snap]. I resent that, often and I find myself not wanting to follow the rules [smile, laughter] but I do, I’m a good interpreter. What I find, is that it’s really a stressor on my mental health, where I have outside factors that are affecting me right now with caregiving and I had a broken leg for the fall – it was just a few things like that. I am finding that my mental health is also compounded with what I’m being expected to do at my job as an interpreter (Interpreter #7). | |
|  | Occupational health is just a piece of the pie of one’s overall health. I really started to be more selective about jobs when I noticed that I was losing it with my kids. I would be so stressed at work. I just didn’t have anything left in my tank for them. They’re really young, it’s stressful anyway. My life situation changing really impacted what jobs I was willing to take because I realized very quickly that I just did not have enough of my tank. I couldn’t do the really stressful work and also be a Mom at the same time or be a decent one. It’s that needing to take care of oneself even when there are those competing demands within you and how you feel about perhaps turning down certain types of work that prioritizing oneself is sometimes something that has to be learned (Interpreter #20). | |
| Notes: ADA=Americans with Disabilities Act; ASL=American Sign Language; BIPOC= black, indigenous, and other people of color; COVID=coronavirus; HR=human resources; IEP=individualized education program; ITP=interpreter training program; LGBTQ=lesbian, gay, bisexual, transgender, and queer or questioning; L2=second language; Q&A=question and answer; RID= Registry of Interpreters for the Deaf; VRI=video remote interpreting; VRS=video relay service | | |
